# Clonal Diversity and Antimicrobial Resistance of Methicillin-Resistant *Staphylococcus pseudintermedius* Isolated from Canine Pyoderma

**DOI:** 10.3390/microorganisms9030482

**Published:** 2021-02-25

**Authors:** Vanessa Silva, Ana Oliveira, Vera Manageiro, Manuela Caniça, Diogo Contente, Rosa Capita, Carlos Alonso-Calleja, Isabel Carvalho, José L. Capelo, Gilberto Igrejas, Patrícia Poeta

**Affiliations:** 1 Microbiology and Antibiotic Resistance Team (MicroART), Department of Veterinary Sciences, University of Trás-os-Montes and Alto Douro (UTAD), 5000-801 Vila Real, Portugal; vanessasilva@utad.pt (V.S.); diogo.contente95@gmail.com (D.C.); isabelcarvalho93@hotmail.com (I.C.); 2 Department of Genetics and Biotechnology, University of Trás-os-Montes and Alto Douro, 5000-801 Vila Real, Portugal; gigrejas@utad.pt; 3 Functional Genomics and Proteomics Unit, University of Trás-os-Montes and Alto Douro (UTAD), 5000-801 Vila Real, Portugal; 4 Associated Laboratory for Green Chemistry (LAQV-REQUIMTE), University NOVA of Lisboa, Lisboa, 2825-466 Caparica, Portugal; 5 Faculty of Veterinary Medicine, University Lusófona de Humanidades e Tecnologias, 1749-024 Lisboa, Portugal; ana.dermatology@gmail.com; 6 National Reference Laboratory of Antibiotic Resistances and Healthcare Associated Infections (NRL-AMR/HAI), Department of Infectious Diseases, National Institute of Health Dr Ricardo Jorge, Av. Padre Cruz, 1649-016 Lisbon, Portugal; vera.manageiro@insa.min-saude.pt (V.M.); manuela.canica@insa.min-saude.pt (M.C.); 7 Centre for the Studies of Animal Science, Institute of Agrarian and Agri-Food Sciences and Technologies, Oporto University, 4051-401 Oporto, Portugal; 8 Department of Food Hygiene and Technology, Veterinary Faculty, University of León, E-24071 León, Spain; rosa.capita@unileon.es (R.C.); carlos.alonso.calleja@unileon.es (C.A.-C.); 9 Institute of Food Science and Technology, University of León, E-24071 León, Spain; 10 BIOSCOPE Group, LAQV@REQUIMTE, Chemistry Department, Faculty of Science and Technology, NOVA University of Lisbon, 2825-466 Almada, Portugal; jlcm@fct.unl.pt; 11 Proteomass Scientific Society, 2825-466 Costa de Caparica, Portugal

**Keywords:** *Staphylococcus pseudintermedius (S. pseudintermedius)*, MRSP, ST123, CC71, multidrug resistance

## Abstract

The emergence of methicillin-resistant *Staphylococcus pseudintermedius* (MRSP) antimicrobial resistance and epidemic genetic lineages is posing a challenge in veterinary medicine due to the limited therapeutical options. MRSP has been identified as an important canine pyoderma pathogen. Thus, we aimed to characterize the antimicrobial resistance and clonal lineages of MRSP isolated from canine cutaneous pyoderma. Thirty-one MRSP isolates recovered from pyoderma were further characterized. The antimicrobial susceptibility testing of the isolates was performed by the Kirby-Bauer disc diffusion method against 14 antimicrobial agents. The presence of antimicrobial and virulence genes was carried out by PCR. Multilocus sequence typing was performed in all isolates. All strains had a multidrug-resistant profile showing resistance mainly to penicillin, macrolides and lincosamides, aminoglycosides, tetracycline and trimethoprim-sulfamethoxazole, which was encoded by the *bla*Z, *erm*B, *msr*(A/B), *aac*(6′)-Ie-*aph*(2′′)-Ia, *aph*(3′)-IIIa, *ant*(4′)-Ia, *tet*M, *tet*K and *dfr*G genes. All isolates harbored the *luk*S-I/*luk*F-I virulence factors. Isolates were ascribed to nine previously described sequence types (STs): ST123, ST339, ST727, ST71, ST537, ST45, ST1029, ST118 and ST1468; and to five STs first described in this study: ST2024, ST2025, ST2026, ST2027 and ST2028. In this study, most isolates belonged to ST123 (n = 16), which belongs to CC71 and is the most common clone in Europe. All isolates were multidrug-resistant, which may impose a serious threat to animal health.

## 1. Introduction

The prevalence of antimicrobial resistant bacteria has been increasing over the years, and it is highly associated with the overuse and misuse of antibiotics in human and veterinary medicine, agriculture and industry [1]. Furthermore, multidrug-resistant bacteria have been increasingly reported as a cause of infections, which makes this issue a major concern in clinical practice worldwide [2]. The indiscriminative use of different antibiotics over the years has led to the emergence of multi-resistant staphylococci strains due to mutations in genes that encode target proteins, and also through the acquisition and accumulation of genes that confer resistance to antibiotics [3]. In general, staphylococci often carry resistance to antibiotics, with resistance to β-lactams such as penicillins, cephalosporins, and carbapenems, being of particular importance [4]. Staphylococci present different mechanisms of resistance to β-lactams, such as the presence of modified penicillin-binding proteins (PBP), the production of β-lactamase enzymes and the tolerance phenomena [5]. Methicillin resistant staphylococci have been recognized as a public health problem and are considered one of the antibiotic-resistant priority pathogens [6]. *Staphylococcus pseudintermedius* is a coagulase-positive species of *staphylococci* that belongs to the *Staphylococcus intermedius* group. *S. pseudintermedius*, similarly to *S. aureus* in humans, colonizes the skin and mucous membranes of some animal species, in particular dogs [7]. Although approximately 90% of healthy dogs are colonized by *S. pseudintermedius*, it is also one of the most common microorganisms causing infection in these animals, especially when the immune system of the host is compromised or if there is a breach in the skin [8,9,10]. While this pathogen is the primary cause of skin and soft tissue infections, it has also been associated with other infections such as external ear otitis, abscess formation, urinary tract infections, mastitis and endocarditis [11]. Indeed, it has been shown that up to 60% of canine cutaneous pyoderma cases are caused by *S. pseudintermedius* [12]. Since 2006, an increasing number of methicillin-resistant *S. pseudintermedius* (MRSP) has been reported in dogs [9]. Studies have reported methicillin resistance rates from 10 to 20% in diseased dogs. However, the rate of methicillin resistance was reported to increase up to 60% in strains isolated from canine pyoderma [13]. The methicillin resistance in *S. pseudintermedius* is conferred by the *mec*A gene, which encodes the penicillin-binding protein 2a (PBP2a) [14]. *S. pseudintermedius* shares some features with *S. aureus*, including some virulence factors. *S. pseudintermedius* produces a bicomponent leukotoxin, similar to Panton-Valentine leukocidin (PVL) from *S. aureus*, which is also encoded by two genes, *luk*S-I and *luk*F-I, that induce cell lysis [15]. Recent studies have shown that MRSP isolated from dogs are multidrug resistant, posing a challenge in veterinary antimicrobial therapy [16]. Several studies have identified geographical patterns of the clonal spread of *S. pseudintermedius*. Multilocus sequence typing has identified several dominant MRSP clones around the world, including ST71 in Europe, ST68 in the United States and ST45/ST112 in Asia [17]. Just like in the rest of Europe, in Portugal, until 2016, the predominant clone of MRSP was also ST71 [18,19]. However, the molecular epidemiology of MRSP is changing in European countries, and more recent studies are required. Thus, in this study, we aimed to characterize the antimicrobial resistance in MRSP isolated from canine pyoderma, as well as the clonal lineages.

## 2. Materials and Methods

### 2.1. Samples and Bacterial Isolates

**Figure 1:**
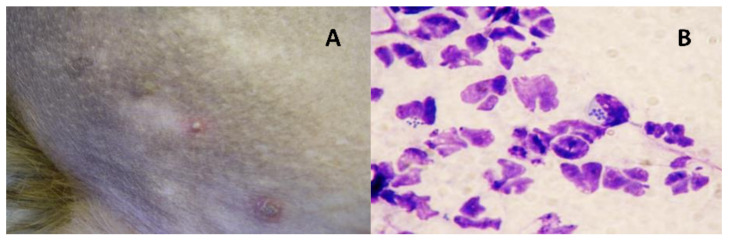
(**A**) Example of a pustule and (**B**) the cytological analysis performed to confirm canine pyoderma.

During the period of one year (from 2019 to 2020), 31 *S. pseudintermedius* were isolated from dogs with cutaneous pyoderma (Figure 1). All dogs considered in this study had previously undergone antibiotic therapy to treat the infection, which was not effective. Isolates were recovered from dogs of different ages (ranging from 2 to 18 years old) and breeds (Table 1). Sterile saline-moistened swabs were used to sample the affected skin. Swabs were inoculated onto 5% Sheep Blood agar and MacConkey agar and incubated aerobically at 37 °C for 24 h. Colonies with morphology characteristic of *S. pseudintermedius* were recovered. The species identification and the presence of the *mec*A gene were confirmed by biochemical tests (Gram staining, catalase and coagulase tests) and by Polymerase Chain Reaction-Restriction Fragment Length Polymorphism (PCR-RFLP) assay [20].

### 2.2. Antimicrobial Susceptibility Testing

Antimicrobial susceptibility testing was performed by the Kirby-Bauer disk diffusion method against 14 antimicrobial agents: penicillin (1 U), cefoxitin (30 μg), ciprofloxacin (5 μg), linezolid (10 µg), gentamicin (10 μg), kanamycin (30 μg), tobramycin (10 μg), erythromycin (15 μg), clindamycin (2 μg), tetracycline (30 μg), chloramphenicol (30 μg), fusidic acid (10 μg), trimethoprim-sulfamethoxazole (1.25/23.75 μg) and mupirocin (200 μg). The interpretation was performed according to the European Committee on Antimicrobial Susceptibility Testing (EUCAST, 2018), except for kanamycin, which followed the Clinical and Laboratory Standards Institute guidelines (CLSI, 2017). *S. aureus* strain ATCC 25923 was used as quality control.

### 2.3. Antibiotic Resistance Genes and Virulence Factors

DNA was extracted from fresh cultures as previously described [21]. Briefly, isolates were seeded onto Brain Heart Infusion agar plates and incubated at 37 °C for 18–24 h. A few colonies of each isolate were suspended in 45 µL of MiliQ-water and 5 µL of lysostaphin (1 mg/mL), and the samples were incubated at 10 min at 37 °C. Then, 45 μL of MilliQ water, 150 µL of Tris-HCl (0.1 M) and 5 μL of proteinase K (2 mg/mL) were added. The samples were then incubated at 67 °C for 10 min and then the samples were boiled for 5 min.

According to the phenotypic resistance of each isolate, the presence of 23 antibiotic resistance genes was studied by PCR using specific primers and conditions as previously described [22]: *bla*Z, *erm*A, *erm*B, *erm*C, *erm*T, *msr*(A/B), *mph*C, *lin*B, *vga*B, *vga*C, *aac*(6′)-Ie-*aph*(2′′)-Ia, *aph*(3′)-IIIa, *ant*(4′)-Ia, *tet*M, *tet*L, *tet*K, *tet*O, *dfr*A, *dfr*D, *dfr*G, *dfr*K, *fus*B and *fus*C.

Virulence factors were determined by PCR with specific primers and conditions as previously described [22,23,24,25,26]: *hla*, *hlb*, *hld*, *tst*, *eta*, *etb*, *luk*S/F-PV and *luk*F/*luk*S-I.

Positive and negative controls used in all experiments belong to the strain collection of the University of Trás-os-Montes and Alto Douro.

### 2.4. Molecular Characterization

Multilocus-sequence-typing (MLST) was performed in all isolates as previously described [27,28]. Sequence types (STs) were assigned based on the *S. pseudintermedius* MLST database (http://pubmlst.org/spseudintermedius accessed on 14 September 2020.).

## 3. Results and Discussion

*S. pseudintermedius* is frequent in dogs and other companion animals and is one of the most frequent causes of cutaneous pyoderma in dogs. Furthermore, the prevalence of MRSP in canine pyoderma is moderate to high according to some studies [29,30]. In this study, samples were collected from 31 dogs with cutaneous pyoderma. All animals were client-owned dogs visiting a Veterinary Hospital in Lisbon, Portugal. Demographic data comprised 16 females and 15 males with ages ranging from 2 to 18 years old. Seven dogs were mixed breed, while the remaining ones belonged to several different breeds (Table 1). However, there seems to be no relation between gender, breed or age and antimicrobial resistance patterns or clonal lineages. In this study, only samples from dogs that had already undergone antibiotic therapy for the treatment of pyoderma but that the treatment was not effective were considered. As expected, all isolates were multidrug-resistant since they showed resistance to at least three antibiotic classes. All isolates harbored the *mec*A gene and were classified as MRSP. The antimicrobial resistance characteristics of the isolates are shown in Table 2. Studies have shown that *S. pseudintermedius* isolates are commonly resistant to penicillins, tetracyclines and macrolides, which are the most frequently used antibiotics in dogs [31]. All MRSP were phenotypically resistant to penicillin and all (except one) harbored the *bla*Z gene. Penicillin resistance in staphylococci is very common and is usually conferred by the staphylococcal beta-lactamase encoded by the *bla*Z gene [32]. Resistance to macrolides and lincosamides was detected in all isolates and was conferred by the genes *erm*B (n = 30), *msr*(A/B) (n = 1) or both (n= 7). Previous studies conducted in Europe, North America and Asia revealed that *erm*B is the predominant erm gene in canine *S. pseudintermedius* [7,29,33]. Resistance to aminoglycosides was observed in all isolates. Yet, unlike what happens in methicillin-resistant *S. aureus* (MRSA) isolates, as most have resistance to gentamicin, in MRSP, resistance to kanamycin seems to be more prevalent than to gentamycin. In our study, all MRSP were phenotypically resistant to kanamycin and all harbored the *aph*(3′)-IIIa gene. However, resistance to gentamicin and tobramycin was observed in 25 and 24 isolates, respectively, of which 20 harbored the bi-funtional enzyme *aac*(6′)-Ie-*aph*(2′′)-Ia and 11 had the *ant*(4′)-Ia gene. Other studies had similar results, with resistance to kanamycin conferred by the *aph*(3′)-IIIa gene as the most prevalent resistance to aminoglycosides in MRSP [34,35]. Resistance to tetracycline in *S. pseudintermedius* and MRSP is common, and is often mediated by *tet*M followed by *tet*K genes [12,36]. In agreement with this, 25 of our isolates showed resistance to tetracycline and harbored either the *tet*M (n = 16) or *tet*K (n = 9) genes. *tet*M codes for ribosome protective proteins, whereas *tet*K codes for efflux pumps [37]. Trimethoprim-sulfamethoxazole resistance was found in 29 isolates. All isolates carried only the *dfr*G gene, which codes for a trimethoprim-resistant dihydrofolate reductase, and this is in accordance with the results of other studies that report the *dfr*G gene as the most common in S. pseudintermedius isolates [7,38]. A high prevalence of dfrG has been found in both MRSP and methicillin-susceptible S. pseudintermedius (MSSP) isolates [36,39]. Previous studies have shown that the vast majority of MRSP isolates often carry a Tn*5405*-like transposable element that carries several antimicrobial resistance genes including *erm*B, *aph*(3′)-IIIa and *dfrG*, which were the most frequently detected genes in this study [39,40]. Phenotypic resistance to ciprofloxacin (n = 24), chloramphenicol (n = 6) and fusidic acid (n = 1) was also detected in our study. Resistance to linezolid and mupirocin was not detected. Regarding the virulence genes, *luk*F-I/*luk*S-I was detected in all isolates; however, none of the other virulence genes tested were detected. These results are in accordance with other studies that obtained a high prevalence of *luk*F-I and *luk*S-I genes in *S. pseudintermedius* canine isolates [19,41,42]. The MRSP isolates were ascribed to nine previously described STs: ST123 (n = 16), ST339 (n = 2), ST727 (n = 2), ST71, ST537, ST45, ST1029, ST118 and ST1468; and to five STs first described in this study: ST2024, ST2025, ST2026, ST2027 and ST2028 (Figure S1). The high genetic diversity is in accordance with other studies conducted in *S. pseudintermedius* [17,43]. ST71 (clonal complex 71) is a widespread clone that has been described as the epidemic European clone. A systematic review on the epidemiology of MRSP conducted in 2016 confirmed that Clonal Complex (CC) 71 was the most reported CC among MRSP in Europe, and that it was only detected in methicillin-resistant strains [44]. More recent studies continue to report CC71 as the predominant clone in MRSP in Italy, Spain and Germany [33,38,45,46]. However, in contrast with these recent studies underlying a higher detection of MRSP belonging to ST71, in our study, only one ST71 was found, with ST123 being the most prevalent clone. Nevertheless, ST123 also belongs to CC71, and it differs from ST71 by a one-point mutation on the *sar* locus. ST123 has been previously described in a study by Duim et al. conducted in MRSP isolated from dogs in the Netherlands in 2016 [47]. Bergot et al. (2018) reported that, although ST71 still remains the most prevalent clone, between 2012–2013 and 2015–2016 the prevalence of ST71 rapidly decreased from 65.3% to 55.2% [17]. In Portugal, in studies performed until 2016, ST71 was also the most prevalent clone [19]. However, the clonal lineages of MRSP isolated from dogs have not been reported since 2016 in this country, and a shift might have occurred. Other plausible explanations may be that the ST123 clone has a higher evolutionary rate or some unknown factor has favored its recent prevalence in canine pyoderma in Portugal. Furthermore, the high occurrence of this clone, closely related to ST71, may be in agreement to Ishihara et al., who stated that the ST71 lineage may not be as clonal as previously believed [48]. ST71 seems to be linked to MRSP, while ST123 has been detected among both MRSP and MSSP [44]. In our study, the ST71 isolate had an extensively drug-resistant profile, showing resistance to penicillin, kanamycin, erythromycin, clindamycin, tetracycline and trimethoprim-sulfamethoxazole. Some studies reported that ST71 typically has tetracycline and chloramphenicol susceptibility [44]. However, the frequency of tetracycline resistance seems to vary geographically in ST71 isolates, and other studies have also found a multidrug-resistant profile, including resistance to tetracycline, in ST71 strains [12,38,46,49]. In our study, among the CC71 isolates, there seems to be a connection between the ST and the resistance profile since, unlike the ST71 isolate, all ST123 strains showed resistance to gentamicin, tobramycin and ciprofloxacin. Two isolates belonged to ST727, which was first described in Sweden and associated with a MRSP isolate according to the *S. pseudintermedius* PubMLST database (http://pubmlst.org/spseudintermedius accessed on 25 January 2021). Two strains belonged to ST339, which is a singleton and has been reported in MRSP from canine pyoderma [14,47]. All of the other STs found in this study belonged to one isolate each. ST1468 was only described in MSSP recovered from a dog nasal swab in a recent study, and ST537 was reported in MRSP from a diseased dog in Australia [7,12]. ST45 is the most prevalent clone in Asia and it is also widely disseminated in Australia. However, it is also relatively common in Europe, often being the second most prevalent clone in dog infections [43,47]. In our study, the ST45 isolate was one of those that showed resistance to more antibiotics, harboring seven antibiotic resistance genes. One isolate belonged to ST1029, which, according to the *S. pseudintermedius* PubMLST database, was first described in MRSP causing canine pyoderma in 2016 in France. Finally, one isolate was ascribed to ST118, which is included in CC258. ST118 has been reported in multiple European countries [44,47].

## 4. Conclusions

In this study, multidrug-resistant MRSP were detected in all isolates and they seem to be a common cause of cutaneous pyoderma, leading to an incidence of subsequent infections. MRSP presented frequent resistance to aminoglycosides, macrolides, tetracycline and trimethoprim-sulfamethoxazole. Given that ST123 was the most prevalent clone detected in this study and has not been previously reported in Portugal, it is likely that it may represent a locally evolved clone. Nevertheless, CC71 clones remain the most common in Europe. Due to the multidrug-resistant profile of MRSP isolated from cutaneous infections, veterinary antibiotic therapy is becoming less effective, imposing a serious threat to animal health.

## Figures and Tables

**Table 1:** Age, gender and breed of dogs included in study.

Isolate	Age (Years)	Gender	Breed
VS2777	5	F	Bull Terrier
VS2778	6	M	Golden Retriever
VS2779	8	F	German Shepherd
VS2780	2	F	Yorkshire Terrier
VS2781	5	M	Labrador Retriever
VS2782	6	M	Boxer
VS2783	10	M	Mixed breed
VS2784	4	M	Estrela Mountain
VS2785	9	F	Mixed breed
VS2786	7	M	Boxer
VS2787	6	M	Golden Retriever
VS2788	6	M	Cocker Spaniel
VS2789	10	M	Rafeiro do Alentejo
VS2790	6	M	Yorkshire Terrier
VS2791	2	M	Mixed breed
VS2792	9	F	French Bulldog
VS2793	12	F	Mixed breed
VS2794	5	F	Bull Terrier
VS2795	7	F	Cocker Spaniel
VS2796	3	F	German Shepherd
VS2797	9	M	Akita
VS2798	4	F	French Bulldog
VS2799	6	M	Spanish Water Dog
VS2800	8	M	Labrador Retriever
VS2801	4	F	Mixed breed
VS2802	5	F	Belgian Shepherd
VS2803	5	M	Beagle
VS2804	4	F	Mixed breed
VS2805	4	F	French Bulldog
VS2806	12	F	Mixed breed
VS2807	18	M	Poodle

**Table 2:** Genetic characteristics of methicillin-resistant *Staphylococcus pseudintermedius* (MRSP) isolated from canine pyoderma in Portugal.

Isolate	Antimicrobial resistance		Virulence	ST (CC)	
	Phenotype	Genotype			
VS2777	PEN-KAN-ERY-CD	*bla*Z, *erm*B, *aph*(3′)-IIIa	*luk*F-I/*luk*S-I	ST2024	
VS2778	PEN-CIP-CN-TOB-KAN-ERY-CD-TET-SXT	*bla*Z, *erm*B, *aph*(3′)-IIIa, *tet*K, *dfr*G	*luk*F-I/*luk*S-I	ST2025	
VS2779	PEN-KAN-ERY-CD-TET-CHL	*bla*Z, *erm*B, *aph*(3′)-IIIa, *tet*M, *dfr*G	*luk*F-I/*luk*S-I	ST2026	
VS2780	PEN-CIP-CN-TOB-KAN-ERY-CD-TET-SXT	*bla*Z, *erm*B, *aph*(3′)-IIIa, *aac*(6′)-Ie-*aph*(2′′)-Ia, *ant*(4′)-Ia, *tet*M, *dfr*G	*luk*F-I/*luk*S-I	ST2027	
VS2781	PEN-CN-TOB-KAN-ERY-CD-TET-SXT	*bla*Z, *erm*B, *aac*(6′)-Ie-*aph*(2′′)-Ia, *tet*M, *dfr*G	*luk*F-I/*luk*S-I	ST2028	
VS2782	PEN-KAN-ERY-CD-TET-SXT	*bla*Z, *erm*B, *aph*(3′)-IIIa, *tet*M, *dfr*G	*luk*F-I/*luk*S-I	ST71 (71)	
VS2783	PEN-CIP-CN-TOB-KAN-ERY-CD-TET-CHL-SXT	*bla*Z, *msr*(A/B), *aph*(3′)-IIIa, *aac*(6′)-Ie-*aph*(2′′)-Ia, *ant*(4′)-Ia, *tet*M, *dfr*G	*luk*F-I/*luk*S-I	ST1468	
VS2784	PEN-CIP-CN-TOB-KAN-ERY-CD-SXT	*bla*Z, *erm*B, *aac*(6′)-Ie-*aph*(2′′)-Ia, *aph*(3′)-IIIa, *ant*(4′)-Ia, *dfr*G	*luk*F-I/*luk*S-I	ST123 (71)	
VS2785	PEN-CIP-KAN-ERY-CD-TET-CHL-SXT	*bla*Z, *erm*B, *tet*M, *dfr*G	*luk*F-I/*luk*S-I	ST727	
VS2786	PEN-CIP-CN-TOB-KAN-ERY-CD-TET-SXT	*bla*Z, *erm*B, *aph*(3′)-IIIa, *tet*M, *dfr*G	*luk*F-I/*luk*S-I	ST339	
VS2787	PEN-CIP-KAN-ERY-CD-SXT	*bla*Z, *erm*B, *aph*(3′)-IIIa, *dfr*G	*luk*F-I/*luk*S-I	ST537	
VS2788	PEN-CIP-KAN-ERY-CD-SXT	*bla*Z, *erm*B, *aph*(3′)-IIIa, *dfr*G	*luk*F-I/*luk*S-I	ST339	
VS2789	PEN-CIP-CN-TOB-KAN-ERY-CD-TET-SXT	*bla*Z, *erm*B, *msr*(A/B), *aph*(3′)-IIIa, *tet*M, *dfr*G	*luk*F-I/*luk*S-I	ST123 (71)	
VS2790	PEN-CIP-CN-TOB-KAN-ERY-CD-TET-CHL-SXT	*bla*Z, *erm*B, *aac*(6′)-Ie-*aph*(2′′)-Ia, *aph*(3′)-IIIa, *tet*M, *dfr*G	*luk*F-I/*luk*S-I	ST123 (71)	
VS2791	PEN-CIP-CN-TOB-KAN-ERY-CD-TET-CHL-SXT	*bla*Z, *erm*B, *msr*(A/B), *aac*(6′)-Ie-*aph*(2′′)-Ia, *aph*(3′)-IIIa, *tet*M, *dfr*G	*luk*F-I/*luk*S-I	ST45 (45)	
VS2792	PEN-CIP-CN-TOB-KAN-ERY-CD-TET-SXT	*bla*Z, *erm*B, *aac*(6′)-Ie-*aph*(2′′)-Ia, *aph*(3′)-IIIa, *ant*(4′)-Ia*, tet*M, *dfr*G	*luk*F-I/*luk*S-I	ST123 (71)	
VS2793	PEN-CIP-CN-TOB-KAN-ERY-CD-TET-SXT	*bla*Z, *erm*B, *aac*(6′)-Ie-*aph*(2′′)-Ia, *aph*(3′)-IIIa, *ant*(4′)-Ia*, tet*M, *dfr*G	*luk*F-I/*luk*S-I	ST123 (71)	
VS2794	PEN-CIP-CN-TOB-KAN-ERY-CD-TET-SXT	*bla*Z, *erm*B, *aac*(6′)-Ie-*aph*(2′′)-Ia, *aph*(3′)-IIIa, *tet*K, *dfr*G	*luk*F-I/*luk*S-I	ST123 (71)	
VS2795	PEN-CIP-CN-TOB-KAN-ERY-CD-TET-SXT	*bla*Z, *erm*B, *aac*(6′)-Ie-*aph*(2′′)-Ia, *aph*(3′)-IIIa, *ant*(4′)-Ia, *tet*M, *dfr*G	*luk*F-I/*luk*S-I	ST123 (71)	
VS2796	PEN-CIP-CN-TOB-KAN-ERY-CD-TET-CHL-SXT	*bla*Z, *erm*B, *aac*(6′)-Ie-*aph*(2′′)-Ia, *aph*(3′)-IIIa, *tet*M, *dfr*G	*luk*F-I/*luk*S-I	ST123 (71)	
VS2797	PEN-CN-TOB-KAN-ERY-CD-TET-SXT-RD	*bla*Z, *erm*B, *aph*(3′)-IIIa, *ant*(4′)-Ia, *tet*K, *dfr*G	*luk*F-I/*luk*S-I	ST1029	
VS2798	PEN-CIP-CN-TOB-KAN-ERY-CD-FD-SXT	*bla*Z, *erm*B, *aac*(6′)-Ie-*aph*(2′′)-Ia, *aph*(3′)-IIIa, *dfr*G	*luk*F-I/*luk*S-I	ST123 (71)	
VS2799	PEN-CN-KAN-ERY-CD-TET-SXT	*bla*Z, *erm*B, *msr*(A/B), *aac*(6′)-Ie-*aph*(2′′)-Ia, *aph*(3′)-IIIa, *tet*K, *dfr*G	*luk*F-I/*luk*S-I	ST727	
VS2800	PEN-CIP-CN-TOB-KAN-ERY-CD-TET-SXT	*bla*Z, *erm*B, *msr*(A/B), *aac*(6′)-Ie-*aph*(2′′)-Ia, *aph*(3′)-IIIa, *ant*(4′)-Ia, *tet*K, *dfr*G	*luk*F-I/*luk*S-I	ST123 (71)	
VS2801	PEN-CIP-CN-TOB-KAN-ERY-CD-TET-SXT	*bla*Z, *erm*B, *msr*(A/B), *aac*(6′)-Ie-*aph*(2′′)-Ia, *aph*(3′)-IIIa, *ant*(4′)-Ia, *tet*K, *dfr*G	*luk*F-I/*luk*S-I	ST123 (71)	
VS2802	PEN-CIP-CN-TOB-KAN-ERY-CD-TET-SXT	*bla*Z, *erm*B, *aac*(6′)-Ie-*aph*(2′′)-Ia, *aph*(3′)-IIIa, *tet*M, *dfr*G	*luk*F-I/*luk*S-I	ST123 (71)	
VS2803	PEN-CIP-CN-TOB-KAN-ERY-CD-TET-SXT	*bla*Z, *erm*B, *aac*(6′)-Ie-*aph*(2′′)-Ia, *aph*(3′)-IIIa, *tet*K, *dfr*G	*luk*F-I/*luk*S-I	ST123 (71)	
VS2804	PEN-CN-TOB-KAN-ERY-CD-TET-SXT	*bla*Z, *erm*B, *msr*(A/B), *aph*(3′)-IIIa, *ant*(4′)-Ia, *tet*K, *dfr*G	*luk*F-I/*luk*S-I	ST118 (258)	
VS2805	PEN-CIP-CN-TOB-KAN-ERY-CD-TET-SXT	*erm*B, *msr*(A/B), *aac*(6′)-Ie-*aph*(2′′)-Ia, *aph*(3′)-IIIa, *tet*K, *dfr*G	*luk*F-I/*luk*S-I	ST123 (71)	
VS2806	PEN-CIP-CN-TOB-KAN-ERY-CD-SXT	*bla*Z, *erm*B, *aac*(6′)-Ie-*aph*(2′′)-Ia, *aph*(3′)-IIIa, *dfr*G	*luk*F-I/*luk*S-I	ST123 (71)	
VS2807	PEN-CIP-CN-TOB-KAN-ERY-CD-TET-SXT	*bla*Z, *erm*B, *aac*(6′)-Ie-*aph*(2′′)-Ia, *aph*(3′)-IIIa, *ant*(4′)-Ia*, tet*M, *dfr*G	*luk*F-I/*luk*S-I	ST123 (71)	

## Supplementary Materials

The following are available online at https://www.mdpi.com/2076-2607/9/3/482/s1, Figure S1: Clonal lineages of MRSP isolated from pyoderma. goeBURST analysis in which the branches are connected with a single locus variant level to show the relation of STs. Red circles indicate STs detected in this study.
