# Supplementary figures and images for: Clonal Diversity and Antimicrobial Resistance of Methicillin-Resistant Staphylococcus pseudintermedius Isolated from Canine Pyoderma
Source: Microorganisms. 2021 Feb 25;9(3):482. doi: 10.3390/microorganisms9030482 (PMC7996521; doi:10.3390/microorganisms9030482)

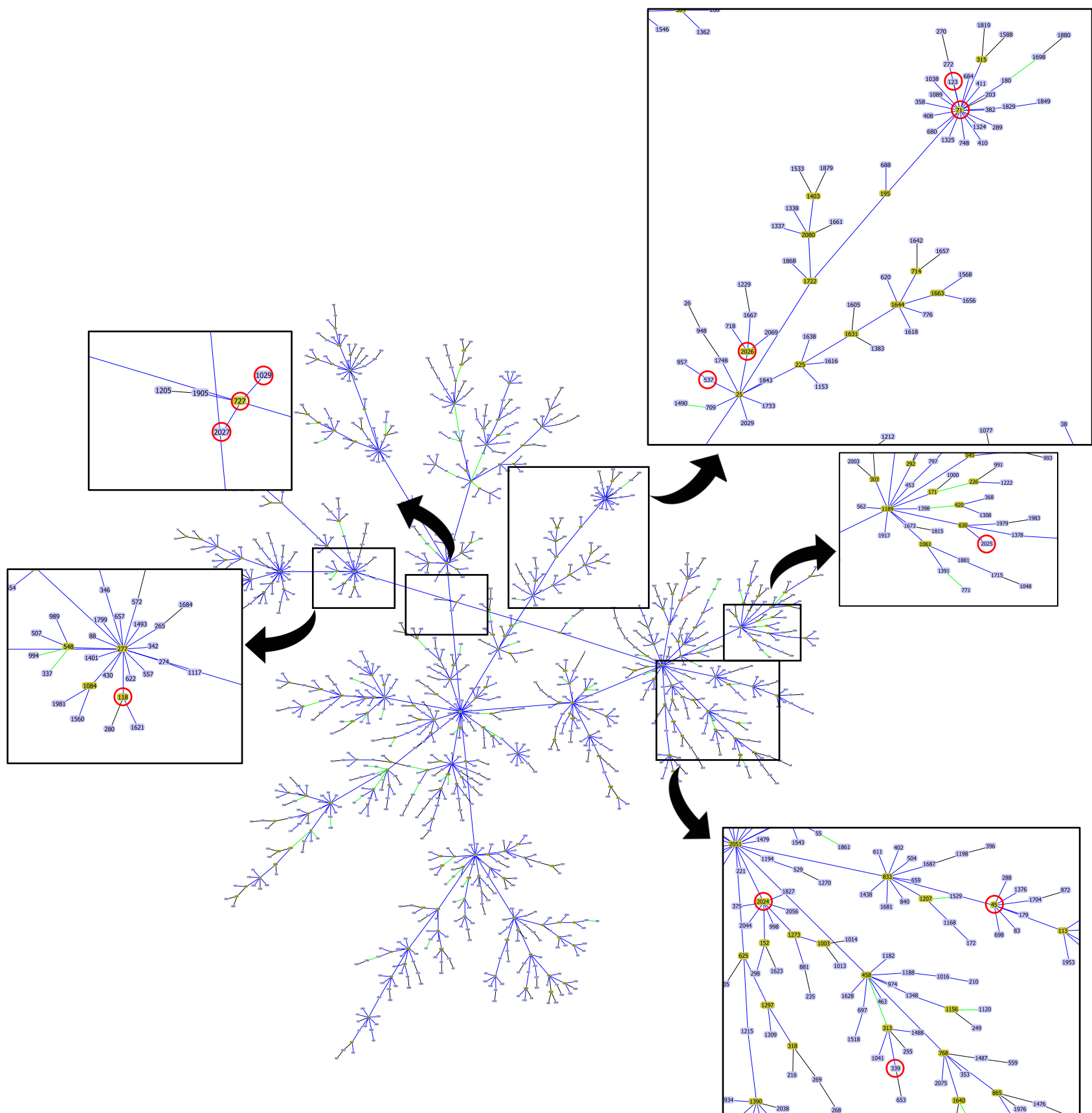

Supplement: Supplementary file 1 [file microorganisms-09-00482-s001.zip › Figure S1 Clonal lineages of MRSP isolated from pyoderma.pdf]
